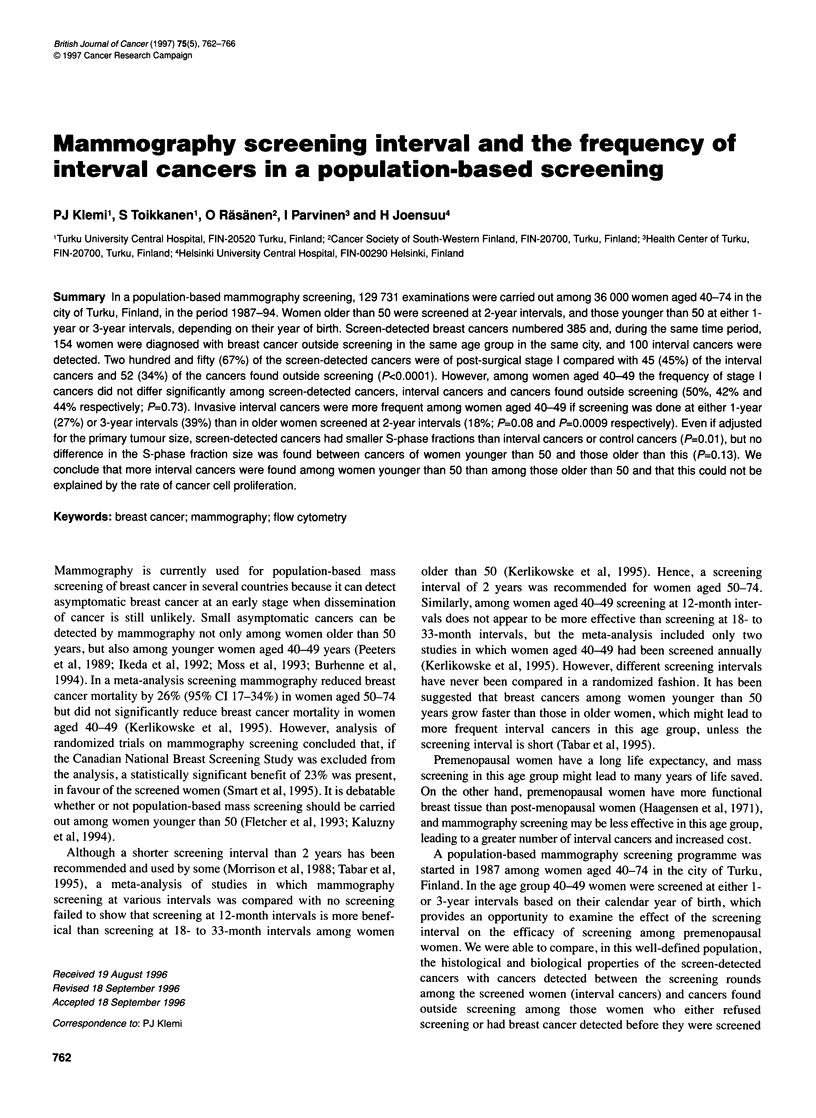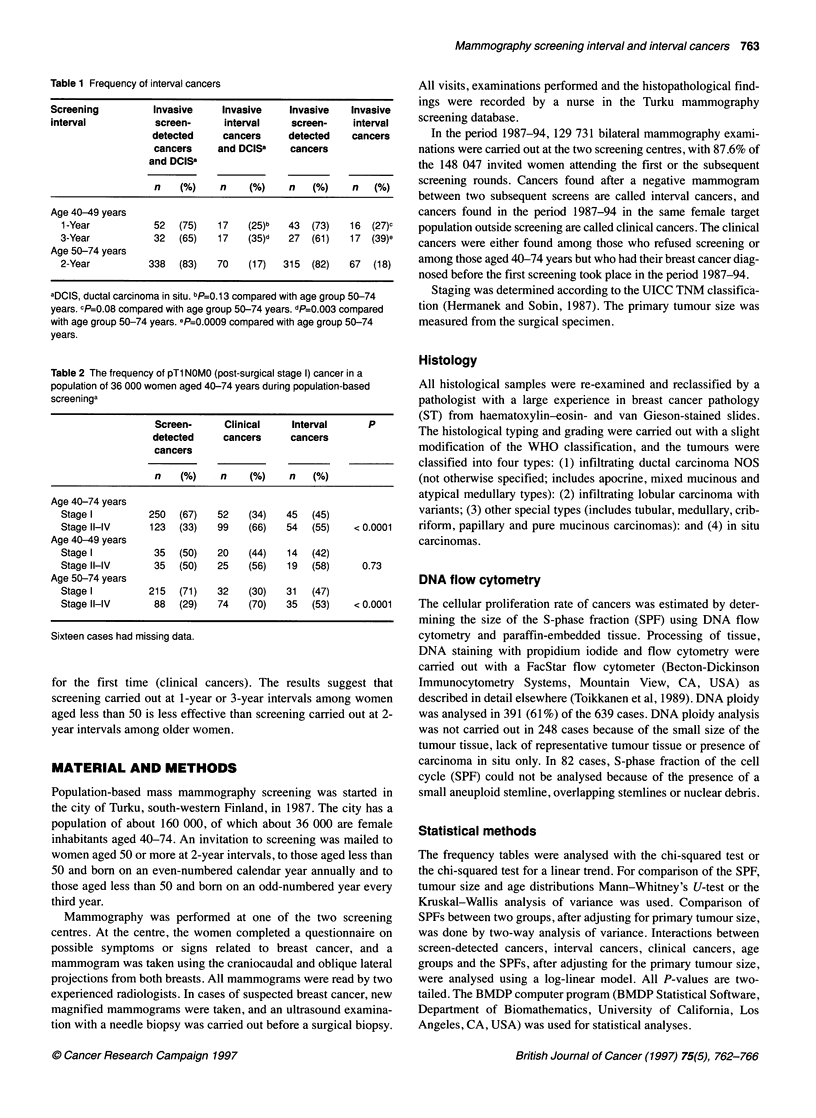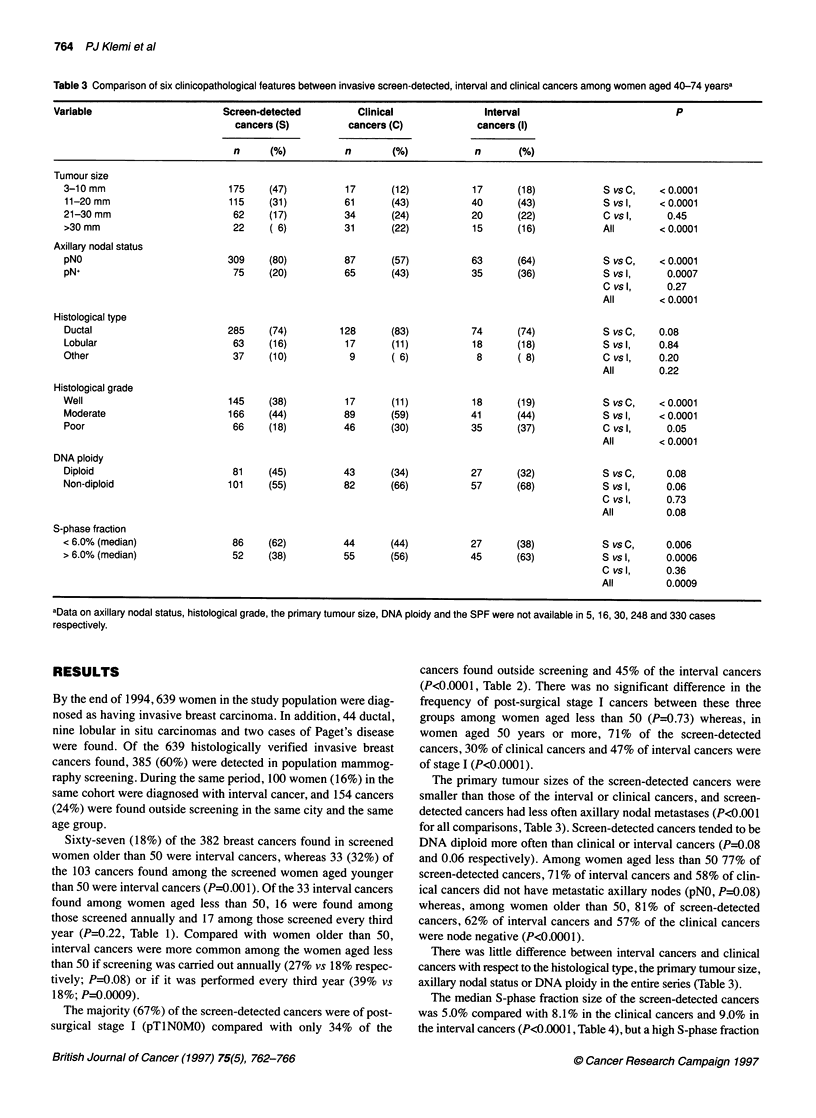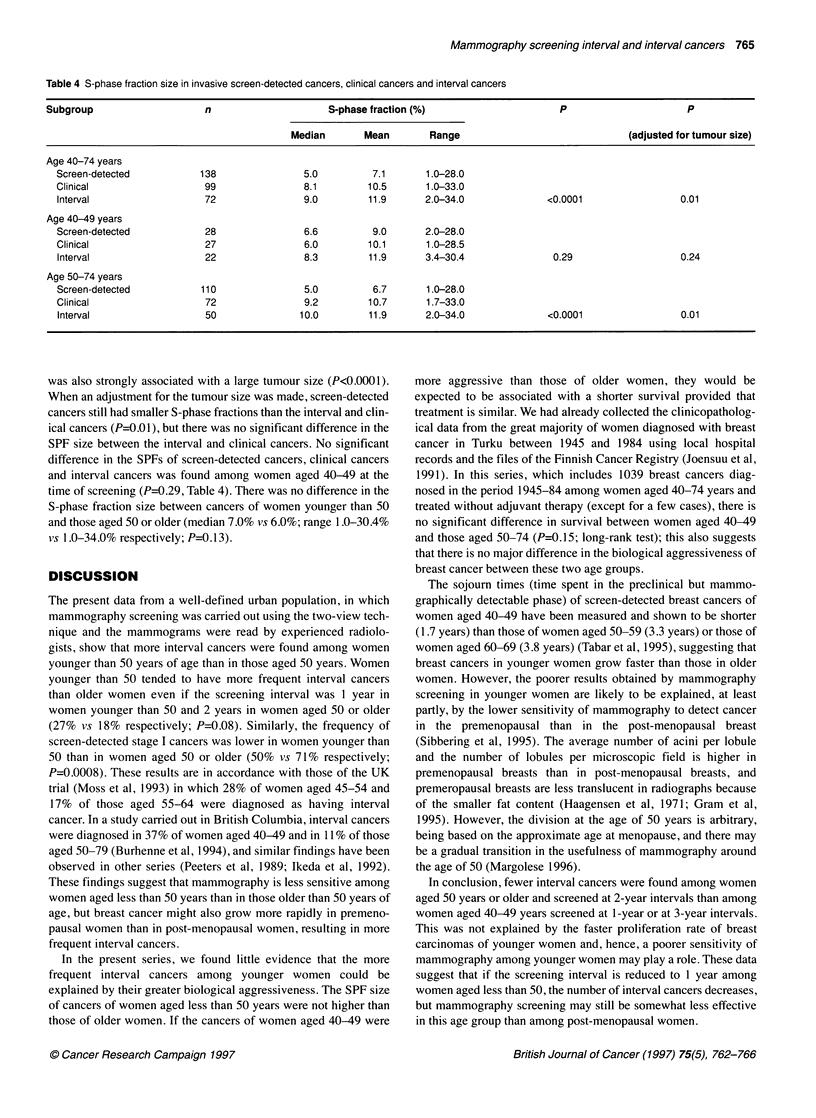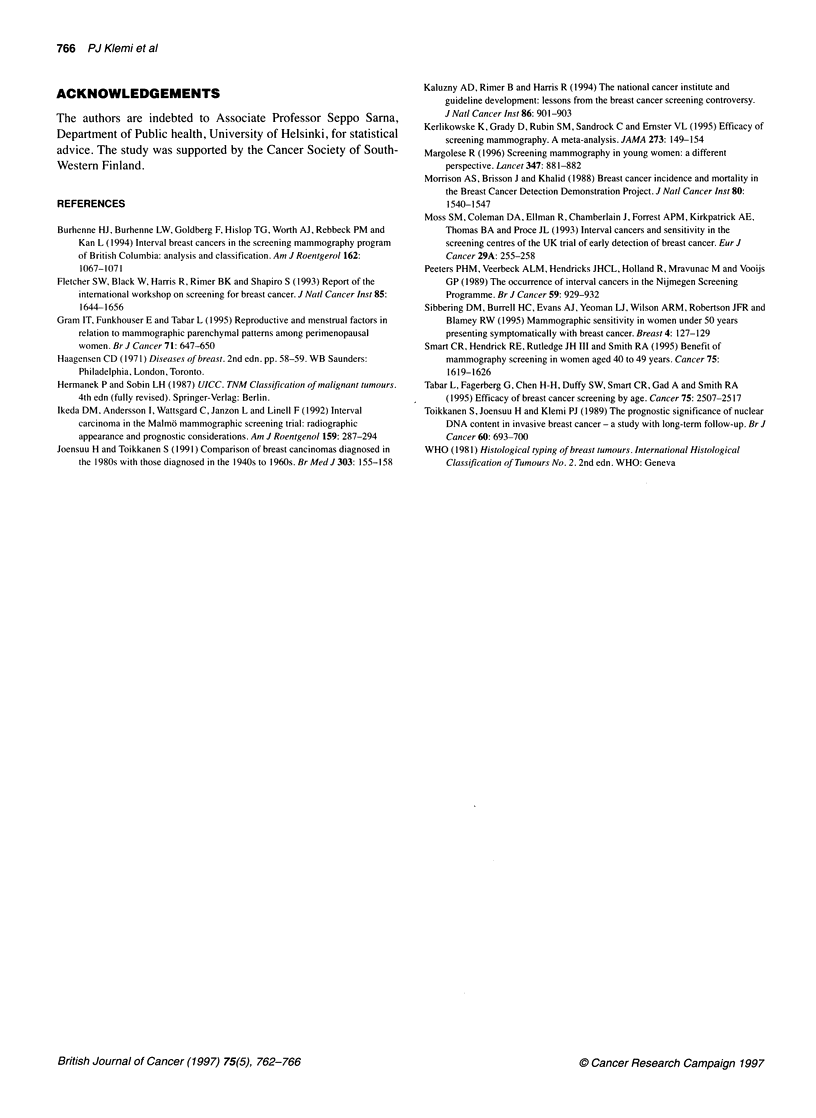# Mammography screening interval and the frequency of interval cancers in a population-based screening.

**DOI:** 10.1038/bjc.1997.135

**Published:** 1997

**Authors:** P. J. Klemi, S. Toikkanen, O. Räsänen, I. Parvinen, H. Joensuu

**Affiliations:** Turku University Central Hospital, Finland.

## Abstract

In a population-based mammography screening, 129,731 examinations were carried out among 36,000 women aged 40-74 in the city of Turku, Finland, in the period 1987-94. Women older than 50 were screened at 2-year intervals, and those younger than 50 at either 1-year or 3-year intervals, depending on their year of birth. Screen-detected breast cancers numbered 385 and, during the same time period, 154 women were diagnosed with breast cancer outside screening in the same age group in the same city, and 100 interval cancers were detected. Two hundred and fifty (67%) of the screen-detected cancers were of post-surgical stage I compared with 45 (45%) of the interval cancers and 52 (34%) of the cancers found outside screening (P<0.0001). However, among women aged 40-49 the frequency of stage I cancers did not differ significantly among screen-detected cancers, interval cancers and cancers found outside screening (50%, 42% and 44% respectively; P=0.73). Invasive interval cancers were more frequent among women aged 40-49 if screening was done at either 1-year (27%) or 3-year intervals (39%) than in older women screened at 2-year intervals (18%; P=0.08 and P=0.0009 respectively). Even if adjusted for the primary tumour size, screen-detected cancers had smaller S-phase fractions than interval cancers or control cancers (P=0.01), but no difference in the S-phase fraction size was found between cancers of women younger than 50 and those older than this (P=0.13). We conclude that more interval cancers were found among women younger than 50 than among those older than 50 and that this could not be explained by the rate of cancer cell proliferation.


British Journal of Cancer (1997) 75(5), 762–766
© 1997 Cancer Research Campaign

### Mammography screening interval and the frequency of interval cancers in a population-based screening

PJ Klemi[1], S Toikkanen[1], O Räsänen[2], I Parvinen[3] and H Joensuu[4]

[1]Turku University Central Hospital, FIN-20520 Turku, Finland; [2]Cancer Society of South-Western Finland, FIN-20700, Turku, Finland; [3]Health Center of Turku, FIN-20700, Turku, Finland; [4]Helsinki University Central Hospital, FIN-00290 Helsinki, Finland

**Summary** In a population-based mammography screening, 129 731 examinations were carried out among 36 000 women aged 40–74 in the city of Turku, Finland, in the period 1987–94. Women older than 50 were screened at 2-year intervals, and those younger than 50 at either 1-year or 3-year intervals, depending on their year of birth. Screen-detected breast cancers numbered 385 and, during the same time period, 154 women were diagnosed with breast cancer outside screening in the same age group in the same city, and 100 interval cancers were detected. Two hundred and fifty (67%) of the screen-detected cancers were of post-surgical stage I compared with 45 (45%) of the interval cancers and 52 (34%) of the cancers found outside screening ($P$<0.0001). However, among women aged 40–49 the frequency of stage I cancers did not differ significantly among screen-detected cancers, interval cancers and cancers found outside screening (50%, 42% and 44% respectively; $P$=0.73). Invasive interval cancers were more frequent among women aged 40–49 if screening was done at either 1-year (27%) or 3-year intervals (39%) than in older women screened at 2-year intervals (18%; $P$=0.08 and $P$=0.0009 respectively). Even if adjusted for the primary tumour size, screen-detected cancers had smaller S-phase fractions than interval cancers or control cancers ($P$=0.01), but no difference in the S-phase fraction size was found between cancers of women younger than 50 and those older than this ($P$=0.13). We conclude that more interval cancers were found among women younger than 50 than among those older than 50 and that this could not be explained by the rate of cancer cell proliferation.

**Keywords:** breast cancer; mammography; flow cytometry

Mammography is currently used for population-based mass screening of breast cancer in several countries because it can detect asymptomatic breast cancer at an early stage when dissemination of cancer is still unlikely. Small asymptomatic cancers can be detected by mammography not only among women older than 50 years, but also among younger women aged 40–49 years (Peeters et al, 1989; Ikeda et al, 1992; Moss et al, 1993; Burhenne et al, 1994). In a meta-analysis screening mammography reduced breast cancer mortality by 26% (95% CI 17–34%) in women aged 50–74 but did not significantly reduce breast cancer mortality in women aged 40–49 (Kerlikowske et al, 1995). However, analysis of randomized trials on mammography screening concluded that, if the Canadian National Breast Screening Study was excluded from the analysis, a statistically significant benefit of 23% was present, in favour of the screened women (Smart et al, 1995). It is debatable whether or not population-based mass screening should be carried out among women younger than 50 (Fletcher et al, 1993; Kaluzny et al, 1994).

Although a shorter screening interval than 2 years has been recommended and used by some (Morrison et al, 1988; Tabar et al, 1995), a meta-analysis of studies in which mammography screening at various intervals was compared with no screening failed to show that screening at 12-month intervals is more benefical than screening at 18- to 33-month intervals among women older than 50 (Kerlikowske et al, 1995). Hence, a screening interval of 2 years was recommended for women aged 50–74. Similarly, among women aged 40–49 screening at 12-month intervals does not appear to be more effective than screening at 18- to 33-month intervals, but the meta-analysis included only two studies in which women aged 40–49 had been screened annually (Kerlikowske et al, 1995). However, different screening intervals have never been compared in a randomized fashion. It has been suggested that breast cancers among women younger than 50 years grow faster than those in older women, which might lead to more frequent interval cancers in this age group, unless the screening interval is short (Tabar et al, 1995).

Premenopausal women have a long life expectancy, and mass screening in this age group might lead to many years of life saved. On the other hand, premenopausal women have more functional breast tissue than post-menopausal women (Haagensen et al, 1971), and mammography screening may be less effective in this age group, leading to a greater number of interval cancers and increased cost.

A population-based mammography screening programme was started in 1987 among women aged 40–74 in the city of Turku, Finland. In the age group 40–49 women were screened at either 1- or 3-year intervals based on their calendar year of birth, which provides an opportunity to examine the effect of the screening interval on the efficacy of screening among premenopausal women. We were able to compare, in this well-defined population, the histological and biological properties of the screen-detected cancers with cancers detected between the screening rounds among the screened women (interval cancers) and cancers found outside screening among those women who either refused screening or had breast cancer detected before they were screened

*Received 19 August 1996*
*Revised 18 September 1996*
*Accepted 18 September 1996*

*Correspondence to*: PJ Klemi

**Table 1** Frequency of interval cancers

| Screening interval | Invasive screen-detected cancers and DCIS[a] | | Invasive interval cancers and DCIS[a] | | Invasive screen-detected cancers | | Invasive interval cancers | |
|---|---|---|---|---|---|---|---|---|
| | *n* | (%) | *n* | (%) | *n* | (%) | *n* | (%) |
| Age 40–49 years | | | | | | | | |
| 1-Year | 52 | (75) | 17 | (25)[b] | 43 | (73) | 16 | (27)[c] |
| 3-Year | 32 | (65) | 17 | (35)[d] | 27 | (61) | 17 | (39)[e] |
| Age 50–74 years | | | | | | | | |
| 2-Year | 338 | (83) | 70 | (17) | 315 | (82) | 67 | (18) |

[a]DCIS, ductal carcinoma in situ. [b]$P$=0.13 compared with age group 50–74 years. [c]$P$=0.08 compared with age group 50–74 years. [d]$P$=0.003 compared with age group 50–74 years. [e]$P$=0.0009 compared with age group 50–74 years.

**Table 2** The frequency of pT1N0M0 (post-surgical stage I) cancer in a population of 36 000 women aged 40–74 years during population-based screening[a]

| | Screen-detected cancers | | Clinical cancers | | Interval cancers | | *P* |
|---|---|---|---|---|---|---|---|
| | *n* | (%) | *n* | (%) | *n* | (%) | |
| Age 40–74 years | | | | | | | |
| Stage I | 250 | (67) | 52 | (34) | 45 | (45) | |
| Stage II–IV | 123 | (33) | 99 | (66) | 54 | (55) | < 0.0001 |
| Age 40–49 years | | | | | | | |
| Stage I | 35 | (50) | 20 | (44) | 14 | (42) | |
| Stage II–IV | 35 | (50) | 25 | (56) | 19 | (58) | 0.73 |
| Age 50–74 years | | | | | | | |
| Stage I | 215 | (71) | 32 | (30) | 31 | (47) | |
| Stage II–IV | 88 | (29) | 74 | (70) | 35 | (53) | < 0.0001 |

Sixteen cases had missing data.

for the first time (clinical cancers). The results suggest that screening carried out at 1-year or 3-year intervals among women aged less than 50 is less effective than screening carried out at 2-year intervals among older women.

### MATERIAL AND METHODS

Population-based mass mammography screening was started in the city of Turku, south-western Finland, in 1987. The city has a population of about 160 000, of which about 36 000 are female inhabitants aged 40–74. An invitation to screening was mailed to women aged 50 or more at 2-year intervals, to those aged less than 50 and born on an even-numbered calendar year annually and to those aged less than 50 and born on an odd-numbered year every third year.

Mammography was performed at one of the two screening centres. At the centre, the women completed a questionnaire on possible symptoms or signs related to breast cancer, and a mammogram was taken using the craniocaudal and oblique lateral projections from both breasts. All mammograms were read by two experienced radiologists. In cases of suspected breast cancer, new magnified mammograms were taken, and an ultrasound examination with a needle biopsy was carried out before a surgical biopsy. All visits, examinations performed and the histopathological findings were recorded by a nurse in the Turku mammography screening database.

In the period 1987–94, 129 731 bilateral mammography examinations were carried out at the two screening centres, with 87.6% of the 148 047 invited women attending the first or the subsequent screening rounds. Cancers found after a negative mammogram between two subsequent screens are called interval cancers, and cancers found in the period 1987–94 in the same female target population outside screening are called clinical cancers. The clinical cancers were either found among those who refused screening or among those aged 40–74 years but who had their breast cancer diagnosed before the first screening took place in the period 1987–94.

Staging was determined according to the UICC TNM classification (Hermanek and Sobin, 1987). The primary tumour size was measured from the surgical specimen.

### Histology

All histological samples were re-examined and reclassified by a pathologist with a large experience in breast cancer pathology (ST) from haematoxylin–eosin- and van Gieson-stained slides. The histological typing and grading were carried out with a slight modification of the WHO classification, and the tumours were classified into four types: (1) infiltrating ductal carcinoma NOS (not otherwise specified; includes apocrine, mixed mucinous and atypical medullary types): (2) infiltrating lobular carcinoma with variants; (3) other special types (includes tubular, medullary, cribriform, papillary and pure mucinous carcinomas): and (4) in situ carcinomas.

### DNA flow cytometry

The cellular proliferation rate of cancers was estimated by determining the size of the S-phase fraction (SPF) using DNA flow cytometry and paraffin-embedded tissue. Processing of tissue, DNA staining with propidium iodide and flow cytometry were carried out with a FacStar flow cytometer (Becton-Dickinson Immunocytometry Systems, Mountain View, CA, USA) as described in detail elsewhere (Toikkanen et al, 1989). DNA ploidy was analysed in 391 (61%) of the 639 cases. DNA ploidy analysis was not carried out in 248 cases because of the small size of the tumour tissue, lack of representative tumour tissue or presence of carcinoma in situ only. In 82 cases, S-phase fraction of the cell cycle (SPF) could not be analysed because of the presence of a small aneuploid stemline, overlapping stemlines or nuclear debris.

### Statistical methods

The frequency tables were analysed with the chi-squared test or the chi-squared test for a linear trend. For comparison of the SPF, tumour size and age distributions Mann–Whitney's $U$-test or the Kruskal–Wallis analysis of variance was used. Comparison of SPFs between two groups, after adjusting for primary tumour size, was done by two-way analysis of variance. Interactions between screen-detected cancers, interval cancers, clinical cancers, age groups and the SPFs, after adjusting for the primary tumour size, were analysed using a log-linear model. All $P$-values are two-tailed. The BMDP computer program (BMDP Statistical Software, Department of Biomathematics, University of California, Los Angeles, CA, USA) was used for statistical analyses.

© Cancer Research Campaign 1997

**Table 3** Comparison of six clinicopathological features between invasive screen-detected, interval and clinical cancers among women aged 40–74 years[a]

| Variable | Screen-detected cancers (S) | | Clinical cancers (C) | | Interval cancers (I) | | *P* | |
|---|---|---|---|---|---|---|---|---|
| | *n* | (%) | *n* | (%) | *n* | (%) | | |
| Tumour size | | | | | | | | |
| 3–10 mm | 175 | (47) | 17 | (12) | 17 | (18) | S *vs* C, | < 0.0001 |
| 11–20 mm | 115 | (31) | 61 | (43) | 40 | (43) | S *vs* I, | < 0.0001 |
| 21–30 mm | 62 | (17) | 34 | (24) | 20 | (22) | C *vs* I, | 0.45 |
| >30 mm | 22 | ( 6) | 31 | (22) | 15 | (16) | All | < 0.0001 |
| Axillary nodal status | | | | | | | | |
| pN0 | 309 | (80) | 87 | (57) | 63 | (64) | S *vs* C, | < 0.0001 |
| pN+ | 75 | (20) | 65 | (43) | 35 | (36) | S *vs* I, | 0.0007 |
| | | | | | | | C *vs* I, | 0.27 |
| | | | | | | | All | < 0.0001 |
| Histological type | | | | | | | | |
| Ductal | 285 | (74) | 128 | (83) | 74 | (74) | S *vs* C, | 0.08 |
| Lobular | 63 | (16) | 17 | (11) | 18 | (18) | S *vs* I, | 0.84 |
| Other | 37 | (10) | 9 | ( 6) | 8 | ( 8) | C *vs* I, | 0.20 |
| | | | | | | | All | 0.22 |
| Histological grade | | | | | | | | |
| Well | 145 | (38) | 17 | (11) | 18 | (19) | S *vs* C, | < 0.0001 |
| Moderate | 166 | (44) | 89 | (59) | 41 | (44) | S *vs* I, | < 0.0001 |
| Poor | 66 | (18) | 46 | (30) | 35 | (37) | C *vs* I, | 0.05 |
| | | | | | | | All | < 0.0001 |
| DNA ploidy | | | | | | | | |
| Diploid | 81 | (45) | 43 | (34) | 27 | (32) | S *vs* C, | 0.08 |
| Non-diploid | 101 | (55) | 82 | (66) | 57 | (68) | S *vs* I, | 0.06 |
| | | | | | | | C *vs* I, | 0.73 |
| | | | | | | | All | 0.08 |
| S-phase fraction | | | | | | | | |
| < 6.0% (median) | 86 | (62) | 44 | (44) | 27 | (38) | S *vs* C, | 0.006 |
| > 6.0% (median) | 52 | (38) | 55 | (56) | 45 | (63) | S *vs* I, | 0.0006 |
| | | | | | | | C *vs* I, | 0.36 |
| | | | | | | | All | 0.0009 |

[a]Data on axillary nodal status, histological grade, the primary tumour size, DNA ploidy and the SPF were not available in 5, 16, 30, 248 and 330 cases respectively.

### RESULTS

By the end of 1994, 639 women in the study population were diagnosed as having invasive breast carcinoma. In addition, 44 ductal, nine lobular in situ carcinomas and two cases of Paget's disease were found. Of the 639 histologically verified invasive breast cancers found, 385 (60%) were detected in population mammography screening. During the same period, 100 women (16%) in the same cohort were diagnosed with interval cancer, and 154 cancers (24%) were found outside screening in the same city and the same age group.

Sixty-seven (18%) of the 382 breast cancers found in screened women older than 50 were interval cancers, whereas 33 (32%) of the 103 cancers found among the screened women aged younger than 50 were interval cancers ($P$=0.001). Of the 33 interval cancers found among women aged less than 50, 16 were found among those screened annually and 17 among those screened every third year ($P$=0.22, Table 1). Compared with women older than 50, interval cancers were more common among the women aged less than 50 if screening was carried out annually (27% *vs* 18% respectively; $P$=0.08) or if it was performed every third year (39% *vs* 18%; $P$=0.0009).

The majority (67%) of the screen-detected cancers were of post-surgical stage I (pT1N0M0) compared with only 34% of the cancers found outside screening and 45% of the interval cancers ($P$<0.0001, Table 2). There was no significant difference in the frequency of post-surgical stage I cancers between these three groups among women aged less than 50 ($P$=0.73) whereas, in women aged 50 years or more, 71% of the screen-detected cancers, 30% of clinical cancers and 47% of interval cancers were of stage I ($P$<0.0001).

The primary tumour sizes of the screen-detected cancers were smaller than those of the interval or clinical cancers, and screen-detected cancers had less often axillary nodal metastases ($P$<0.001 for all comparisons, Table 3). Screen-detected cancers tended to be DNA diploid more often than clinical or interval cancers ($P$=0.08 and 0.06 respectively). Among women aged less than 50 77% of screen-detected cancers, 71% of interval cancers and 58% of clinical cancers did not have metastatic axillary nodes (pN0, $P$=0.08) whereas, among women older than 50, 81% of screen-detected cancers, 62% of interval cancers and 57% of the clinical cancers were node negative ($P$<0.0001).

There was little difference between interval cancers and clinical cancers with respect to the histological type, the primary tumour size, axillary nodal status or DNA ploidy in the entire series (Table 3).

The median S-phase fraction size of the screen-detected cancers was 5.0% compared with 8.1% in the clinical cancers and 9.0% in the interval cancers ($P$<0.0001, Table 4), but a high S-phase fraction

© Cancer Research Campaign 1997

**Table 4** S-phase fraction size in invasive screen-detected cancers, clinical cancers and interval cancers

| **Subgroup** | ***n*** | **S-phase fraction (%)** | | | ***P*** | ***P*** |
|---|---|---|---|---|---|---|
| | | **Median** | **Mean** | **Range** | | **(adjusted for tumour size)** |
| Age 40–74 years | | | | | | |
| Screen-detected | 138 | 5.0 | 7.1 | 1.0–28.0 | | |
| Clinical | 99 | 8.1 | 10.5 | 1.0–33.0 | | |
| Interval | 72 | 9.0 | 11.9 | 2.0–34.0 | <0.0001 | 0.01 |
| Age 40–49 years | | | | | | |
| Screen-detected | 28 | 6.6 | 9.0 | 2.0–28.0 | | |
| Clinical | 27 | 6.0 | 10.1 | 1.0–28.5 | | |
| Interval | 22 | 8.3 | 11.9 | 3.4–30.4 | 0.29 | 0.24 |
| Age 50–74 years | | | | | | |
| Screen-detected | 110 | 5.0 | 6.7 | 1.0–28.0 | | |
| Clinical | 72 | 9.2 | 10.7 | 1.7–33.0 | | |
| Interval | 50 | 10.0 | 11.9 | 2.0–34.0 | <0.0001 | 0.01 |

was also strongly associated with a large tumour size ($P$<0.0001). When an adjustment for the tumour size was made, screen-detected cancers still had smaller S-phase fractions than the interval and clinical cancers ($P$=0.01), but there was no significant difference in the SPF size between the interval and clinical cancers. No significant difference in the SPFs of screen-detected cancers, clinical cancers and interval cancers was found among women aged 40–49 at the time of screening ($P$=0.29, Table 4). There was no difference in the S-phase fraction size between cancers of women younger than 50 and those aged 50 or older (median 7.0% *vs* 6.0%; range 1.0–30.4% *vs* 1.0–34.0% respectively; $P$=0.13).

### DISCUSSION

The present data from a well-defined urban population, in which mammography screening was carried out using the two-view technique and the mammograms were read by experienced radiologists, show that more interval cancers were found among women younger than 50 years of age than in those aged 50 years. Women younger than 50 tended to have more frequent interval cancers than older women even if the screening interval was 1 year in women younger than 50 and 2 years in women aged 50 or older (27% *vs* 18% respectively; $P$=0.08). Similarly, the frequency of screen-detected stage I cancers was lower in women younger than 50 than in women aged 50 or older (50% *vs* 71% respectively; $P$=0.0008). These results are in accordance with those of the UK trial (Moss et al, 1993) in which 28% of women aged 45–54 and 17% of those aged 55–64 were diagnosed as having interval cancer. In a study carried out in British Columbia, interval cancers were diagnosed in 37% of women aged 40–49 and in 11% of those aged 50–79 (Burhenne et al, 1994), and similar findings have been observed in other series (Peeters et al, 1989; Ikeda et al, 1992). These findings suggest that mammography is less sensitive among women aged less than 50 years than in those older than 50 years of age, but breast cancer might also grow more rapidly in premenopausal women than in post-menopausal women, resulting in more frequent interval cancers.

In the present series, we found little evidence that the more frequent interval cancers among younger women could be explained by their greater biological aggressiveness. The SPF size of cancers of women aged less than 50 years were not higher than those of older women. If the cancers of women aged 40–49 were more aggressive than those of older women, they would be expected to be associated with a shorter survival provided that treatment is similar. We had already collected the clinicopathological data from the great majority of women diagnosed with breast cancer in Turku between 1945 and 1984 using local hospital records and the files of the Finnish Cancer Registry (Joensuu et al, 1991). In this series, which includes 1039 breast cancers diagnosed in the period 1945–84 among women aged 40–74 years and treated without adjuvant therapy (except for a few cases), there is no significant difference in survival between women aged 40–49 and those aged 50–74 ($P$=0.15; long-rank test); this also suggests that there is no major difference in the biological aggressiveness of breast cancer between these two age groups.

The sojourn times (time spent in the preclinical but mammographically detectable phase) of screen-detected breast cancers of women aged 40–49 have been measured and shown to be shorter (1.7 years) than those of women aged 50–59 (3.3 years) or those of women aged 60–69 (3.8 years) (Tabar et al, 1995), suggesting that breast cancers in younger women grow faster than those in older women. However, the poorer results obtained by mammography screening in younger women are likely to be explained, at least partly, by the lower sensitivity of mammography to detect cancer in the premenopausal than in the post-menopausal breast (Sibbering et al, 1995). The average number of acini per lobule and the number of lobules per microscopic field is higher in premenopausal breasts than in post-menopausal breasts, and premeropausal breasts are less translucent in radiographs because of the smaller fat content (Haagensen et al, 1971; Gram et al, 1995). However, the division at the age of 50 years is arbitrary, being based on the approximate age at menopause, and there may be a gradual transition in the usefulness of mammography around the age of 50 (Margolese 1996).

In conclusion, fewer interval cancers were found among women aged 50 years or older and screened at 2-year intervals than among women aged 40–49 years screened at 1-year or at 3-year intervals. This was not explained by the faster proliferation rate of breast carcinomas of younger women and, hence, a poorer sensitivity of mammography among younger women may play a role. These data suggest that if the screening interval is reduced to 1 year among women aged less than 50, the number of interval cancers decreases, but mammography screening may still be somewhat less effective in this age group than among post-menopausal women.

© Cancer Research Campaign 1997

### ACKNOWLEDGEMENTS

The authors are indebted to Associate Professor Seppo Sarna, Department of Public health, University of Helsinki, for statistical advice. The study was supported by the Cancer Society of South-Western Finland.

### REFERENCES

Burhenne HJ, Burhenne LW, Goldberg F, Hislop TG, Worth AJ, Rebbeck PM and Kan L (1994) Interval breast cancers in the screening mammography program of British Columbia: analysis and classification. *Am J Roentgerol* **162**: 1067–1071

Fletcher SW, Black W, Harris R, Rimer BK and Shapiro S (1993) Report of the international workshop on screening for breast cancer. *J Natl Cancer Inst* **85**: 1644–1656

Gram IT, Funkhouser E and Tabar L (1995) Reproductive and menstrual factors in relation to mammographic parenchymal patterns among perimenopausal women. *Br J Cancer* **71**: 647–650

Haagensen CD (1971) *Diseases of breast*. 2nd edn. pp. 58–59. WB Saunders: Philadelphia, London, Toronto.

Hermanek P and Sobin LH (1987) *UICC. TNM Classification of malignant tumours*. 4th edn (fully revised). Springer-Verlag: Berlin.

Ikeda DM, Andersson I, Wattsgard C, Janzon L and Linell F (1992) Interval carcinoma in the Malmö mammographic screening trial: radiographic appearance and prognostic considerations. *Am J Roentgenol* **159**: 287–294

Joensuu H and Toikkanen S (1991) Comparison of breast cancinomas diagnosed in the 1980s with those diagnosed in the 1940s to 1960s. *Br Med J* **303**: 155–158

Kaluzny AD, Rimer B and Harris R (1994) The national cancer institute and guideline development: lessons from the breast cancer screening controversy. *J Natl Cancer Inst* **86**: 901–903

Kerlikowske K, Grady D, Rubin SM, Sandrock C and Ernster VL (1995) Efficacy of screening mammography. A meta-analysis. *JAMA* **273**: 149–154

Margolese R (1996) Screening mammography in young women: a different perspective. *Lancet* **347**: 881–882

Morrison AS, Brisson J and Khalid (1988) Breast cancer incidence and mortality in the Breast Cancer Detection Demonstration Project. *J Natl Cancer Inst* **80**: 1540–1547

Moss SM, Coleman DA, Ellman R, Chamberlain J, Forrest APM, Kirkpatrick AE, Thomas BA and Proce JL (1993) Interval cancers and sensitivity in the screening centres of the UK trial of early detection of breast cancer. *Eur J Cancer* **29A**: 255–258

Peeters PHM, Veerbeck ALM, Hendricks JHCL, Holland R, Mravunac M and Vooijs GP (1989) The occurrence of interval cancers in the Nijmegen Screening Programme. *Br J Cancer* **59**: 929–932

Sibbering DM, Burrell HC, Evans AJ, Yeoman LJ, Wilson ARM, Robertson JFR and Blamey RW (1995) Mammographic sensitivity in women under 50 years presenting symptomatically with breast cancer. *Breast* **4**: 127–129

Smart CR, Hendrick RE, Rutledge JH III and Smith RA (1995) Benefit of mammography screening in women aged 40 to 49 years. *Cancer* **75**: 1619–1626

Tabar L, Fagerberg G, Chen H-H, Duffy SW, Smart CR, Gad A and Smith RA (1995) Efficacy of breast cancer screening by age. *Cancer* **75**: 2507–2517

Toikkanen S, Joensuu H and Klemi PJ (1989) The prognostic significance of nuclear DNA content in invasive breast cancer – a study with long-term follow-up. *Br J Cancer* **60**: 693–700

WHO (1981) *Histological typing of breast tumours. International Histological Classification of Tumours No. 2*. 2nd edn. WHO: Geneva

© Cancer Research Campaign 1997